# Design and Preparation of White High-Strength Concrete with Ground Limestone Powder by Means of Response Surface Methodology

**DOI:** 10.3390/ma15093359

**Published:** 2022-05-07

**Authors:** Jingliang Xia, Changwei Cao, Zhengwu Jiang, Qiang Ren, Ying Zhang, Jing Wang, Faguang Leng

**Affiliations:** 1 Key Laboratory of Advanced Civil Engineering Materials of Ministry of Education, School of Materials Science and Engineering, Tongji University, Shanghai 201804, China; xiajingliang@cabrtech.com (J.X.); renqiang@tongji.edu.cn (Q.R.); 2 Institute of Building Materials, China Academy of Building Research, Beijing 100013, China; zhangying3@cabrtech.com (Y.Z.); wangjing@cabrtech.com (J.W.); lengfaguang@126.com (F.L.); 3 China Road and Bridge Engineering Co., Ltd., Beijing 100011, China; caow@crbc.com

**Keywords:** response surface methodology, white high-strength concrete, whiteness, multi-objective optimization

## Abstract

This paper investigates the properties of white high-strength concrete (WHSC) prepared with ground limestone powder (GLP). Response surface methodology (RSM) was used to design the proportions of mixes and evaluate the influence of the water–binder ratio (w/b), slurry volume fraction (Vs), and the content of GLP in a binder (Cg) on the slump, whiteness and compressive strength of WHSC via Box–Behnken equations. Results indicate that quadratic polynomial regression equations can be used to predict the performance of WHSC as influenced by combined factors. Both slump and compressive strength of WHSC are found highly influenced by w/b while GLP significantly improves the whiteness of WHSC. An optimal mix proportion of WHSC is provided by the multi-objective optimization with high-accuracy predictions. This paper demonstrates the feasibility of preparing WHSC with GLP and presents the potential of using RSM in the mix proportioning of concrete.

## 1. Introduction

White concrete is attracting increasing attention from architects worldwide [1,2,3,4]. However, the main binder of white concrete is white Portland cement, making it much more expensive than ordinary concrete. This limits the utility of white concrete in engineering applications. Thus, many studies have been performed to reduce the cost of white concrete without significantly sacrificing performance. It was found by Lübeck et al. [5] that white concrete with 50% white Portland cement replaced by slag presented favorable compressive strength and the same white value compared to that with pure white Portland cement. In addition to slag, other solid waste powders such as rice husk were also used to prepare white concrete. It was demonstrated by Ferraro et al. [6] that off-white rice husk ash provided favorable white value and improved compressive strength and durability. In addition, Çolak [7] evaluated the usage of limestone powder in white concrete and found the decreased wear resistance of white concrete with limestone powder.

With the increasing shortage of river sand, manufactured sand, especially limestone manufactured sand, has been gradually taken as a main fine aggregate for concrete [8,9]. Normally, more than 20% limestone powder is produced simultaneously with the production of crushed sand, depending on the crushing craft and rock lithology [10]. However, only limited content of limestone powder can be left in the sand because of the concrete properties lost with excessive limestone powder [11]. As a result, limestone powder yield is increasing in addition to that produced by traditional limestone quarry industries. Increasing attention is attracted to increasing the usage of limestone powder as the mineral admixture of concrete.

Grinding has been proven as an effective solution to improve the properties of limestone powder. It is found that ground stone powder can improve the working performance and strength of concrete compared with ordinary stone powder [12]. However, ground limestone powder (GLP) is normally used in concrete with normal strength, while there is limited literature concerning the usage of limestone powder in high-strength concrete. In addition to the improvement of mechanical properties, grinding also provides a whiteness value greater than 95, meaning GLP has a high potential to be used in white concrete.

However, the interactions between multiple parameters and properties make it a challenge to identify the optimal mix proportion from simple orthogonal experiments, as a large number of samples are required. From this viewpoint, numerical methods can be used for analyzing the covered relationship between properties and mix proportions. Response surface methodology (RSM) has been one of the most useful tools to explain and establish the mathematical relation between input variables and the output responses [13]. It involves the use of experiments designed to collect sufficient data to enable the exploration of the relationships between several explanatory variables and one or more response variables using multiple quadratic regression equations. RSM has been widely applied to identify the best designs in many fields, such as oil and food production, chemical engineering, and biological engineering [14,15,16,17,18,19,20]. However, few reports can be found using RSM for the analyses of concrete properties. One of the advantages of RSM is that it can be used to optimize some of the responses by setting out a series of criteria for each of the independent variables, showing the potential for the optimization of mix proportions of white high-strength concrete (WHSC) with GLP.

Therefore, this paper investigates the potential of GLP as the mineral admixture to prepare white high-strength concrete. The influence of the water–binder ratio (w/b), the slurry volume fraction (V_s_), and the content of GLP in a binder (C_g_) on the workability, whiteness, and compressive strength of WHSCs were analyzed via RSM. In addition, the optimal mix proportion is predicted and validated.

## 2. Materials and methods

### 2.1. Materials

**Figure 1:**
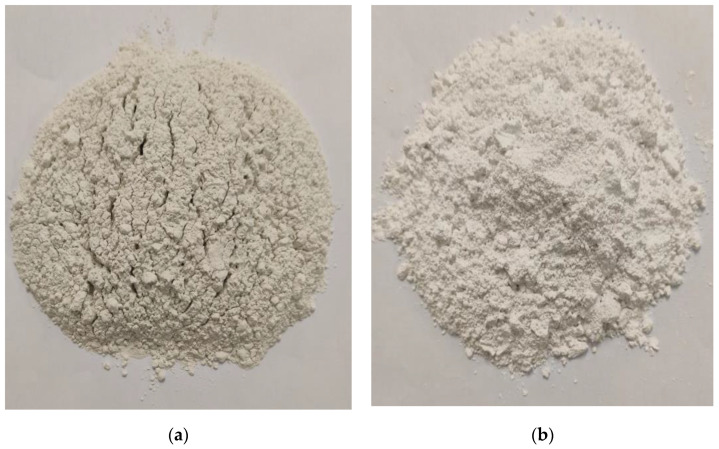
White Portland cement powder (**a**) and GLP (**b**).

**Figure 2:**
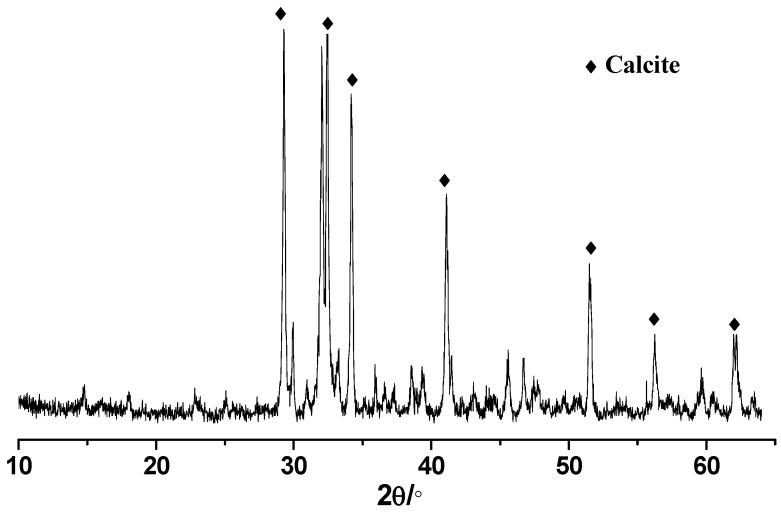
XRD pattern of GLP.

**Figure 3:**
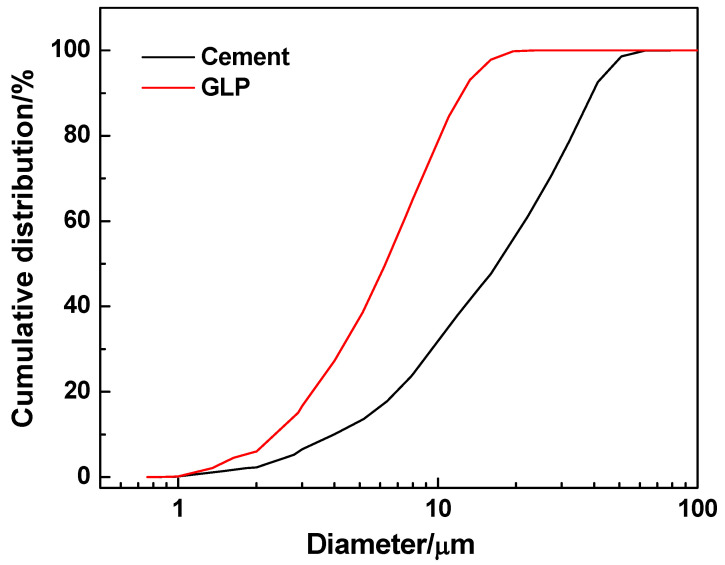
The particle size distributions of cement and GLP.

CEM Ⅰ 52.5 white Portland cement was used (Figure 1), with its physical properties and chemical composition shown in Table 1 and Table 2, respectively. The GLP used in the study has a specific surface area of 850 m^2^/kg, a whiteness of 97.2, and a density of 2.61 g/cm^3^. The chemical composition of GLP is given in Table 2 with the X-ray diffraction pattern shown in Figure 2. It is obvious that the main composition of GLP is calcite. The particle size distributions of cement and GLP are shown in Figure 3.

A high-performance water-reducing agent was used (Sika polycarboxylic acid), with a solid content of 21.3% and a water-reducing rate of 27.5%.

River sand with a fineness modulus of 2.5 and a density of 2.62 g/cm^3^ was used as the fine aggregate. Crushed gneiss with a diameter between 5 mm and 25 mm was used as the coarse aggregate.

### 2.2. Mix Proportions and Sample Preparation

Different model types exist in the RSM analysis package, including Box–Behnken, Central Composite, historical, and so on. In Box–Behnken design, factors are usually taken at three levels and all the design points fall within the safe operating zone. It is considered to be more proficient and powerful than other designs. In addition, it is typical for three input variables, which is the case in the current study. Thus, it is used for experimental design and data analysis. The independent variables used are w/b, Vs, and C_g_, represented by factors A, B, and C, respectively. Factors and factor levels of independent variables are shown in Table 3. The Design-Expert 13 software (Stat-Ease, Inc., Minneapolis, MN, USA) generated a total of 17 mixes using combinations of the independent variables as shown in Table 4. The fraction of sand in aggregate in all mixtures was fixed at 42% in mass. The dosage of water-reducing agent was fixed at 1% of cementitious material in mass.

### 2.3. Testing Methods

#### 2.3.1. Workability

The workability of fresh concrete mixtures was measured via the slump test according to the Chinese national standard GB/T50080-2016 [21].

#### 2.3.2. Compressive Strength

The compressive strength of hardened concrete mixtures was tested according to the Chinese national standard GB/T50081-2019 [22].

#### 2.3.3. Whiteness

**Figure 4:**
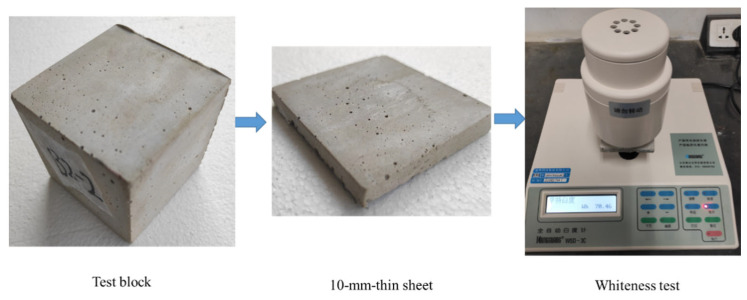
Protocol of testing the whiteness of concrete.

Whiteness was measured according to the Chinese national standard GB/T 5950-2008 [23]. Briefly, a cubic concrete was prepared and cured for 28 days. Then, it was dried and cut into thin sheets with a thickness of 10 ± 1 mm. The whiteness of these sheets was then measured using a whiteness meter (SC-80C). The operational process is shown in Figure 4.

#### 2.3.4. Micro Morphology

The micro morphology of the hardened mixtures at 3 days and 28 days was observed by a scanning electron microscope (ISI-SX-40, ISI, Japan) with an accelerated beam current of 80 mA and a voltage of 20 kV.

## 3. Statistical Modelling Using Box–Behnken Design

### 3.1. Test Results and Model Analysis

The slump, whiteness, and 28-day compressive strength of the WHSCs formed from the mixing proportions defined in Table 4 are shown in Table 5, together with the values predicted by the RSM-based Box–Behnken analyses. RSM was used to perform statistical modeling for predicting the slump, whiteness, and compressive strength of WHSC. Analysis of variance (ANOVA) was used to evaluate each model for the slump, whiteness, and compressive strength shown in Table 6, Table 7 and Table 8, respectively.

The statistical significance of the hypothesis testing analysis and mismatch test analysis is expressed by *p*. The smaller the probability (*p*-value), the more significant the model is. Specifically, *p* < 0.01, 0.01 < *p* < 0.05, and *p* > 0.05 represent very significant, significant, and not significant, respectively. It can be seen from Table 6 that the sequential *p*-values of the quadratic polynomial model are the smallest while the corresponding mismatched *p*-value, adjusted value of the coefficient of determination (*R*^2^), and predicted value of *R*^2^ are the largest. Similar results can be found for both whiteness and compressive strength. Therefore, the quadratic polynomial model is used in the modeling.

### 3.2. Establishment of Regression Equation and Optimization Test

The response surface Box–Behnken analysis was used to perform a multiple regression fitting of the test data in Table 5. The regression models of the slump (Y_1_), whiteness (Y_2_), and compressive strength (Y_3_) are shown in Equations (1)–(3), respectively.

Y_1_ = 214 + 6.25A + 1.875B + 6.875C − 2.5AB + 1.25BC − 7.625A^2^ − 6.375B^2^ − 6.375C^2^ (1)

Y_2_ = 78.4 + 0.95A + 1.0375B + 5.1125C − 0.325AB − 2.225AC + 0.35BC − 2.4A^2^ − 1.125B^2^ − 1.275C^2^ (2)

Y_3_ = 73.96 − 6.0875A + 0.9625B − 1.375C − 0.425AB − 2.4AC − 1.4BC − 2.6175A^2^ + 1.3825B^2^ − 0.7424C^2^ (3)

Table 9 lists the ANOVA results for the above simulation equations, where F is a significant test indicator. The coexistence of a larger F value and a smaller *p*-value indicates that the model has a stronger significance and a higher simulation accuracy. The *p*-value of the mismatch term reflects the significance of the non-correlation between the test data and the model. When *p* < 0.05, i.e., the 95% confidence interval, the significance is high. Table 9 shows that the *p* values of the concrete slump (Y_1_), whiteness (Y_2_), and compressive strength (Y_3_) models are 0.0152, 0.0039, and 0.0155, respectively, and the F values are 5.79, 9.22, and 5.76, respectively. Thus, these values are significant, and the whiteness and compressive strength values are very significant. Among the three single factors, w/b (A) and C_g_ (C) are significant while Vs (B) is not significant. Additionally, the order of influence on the slump is C > A > B. Similarly, it is also found that the whiteness of concrete is significantly influenced by C but not A or B. The order of influence on concrete whiteness is C > B > A. In addition, the compressive strength is significantly influenced by A, but not by B or C. The order of influence on compressive strength is A > C > B.

The closeness of the adjusted and predicted *R*^2^ values can be used to verify the fitting degree of a regression equation. In addition, the smaller the value for the coefficient of variation (CV), the higher the reliability and accuracy of a test for the signal-to-noise ratio greater than four. Table 10 shows the ANOVA results for developed response models. It is seen the signal-to-noise ratios are 6.811, 11.285, and 9.264 for predictions of Y_1,_ Y_2,_ and Y_3_, respectively. In addition, the *R*^2^ values of predictions for Y_1_, Y_2,_ and Y_3_ are 0.8816, 0.9222, and 0.8810, respectively. The adjusted values of *R*^2^ are 0.7294, 0.8221, and 0.7280, respectively, while the CVs are 2.54%, 2.44%, and 3.75%, respectively. It is thus inferred from the above data that the developed models have high reliability and accuracy.

## 4. Results and Discussion

### 4.1. Slump

**Figure 5:**
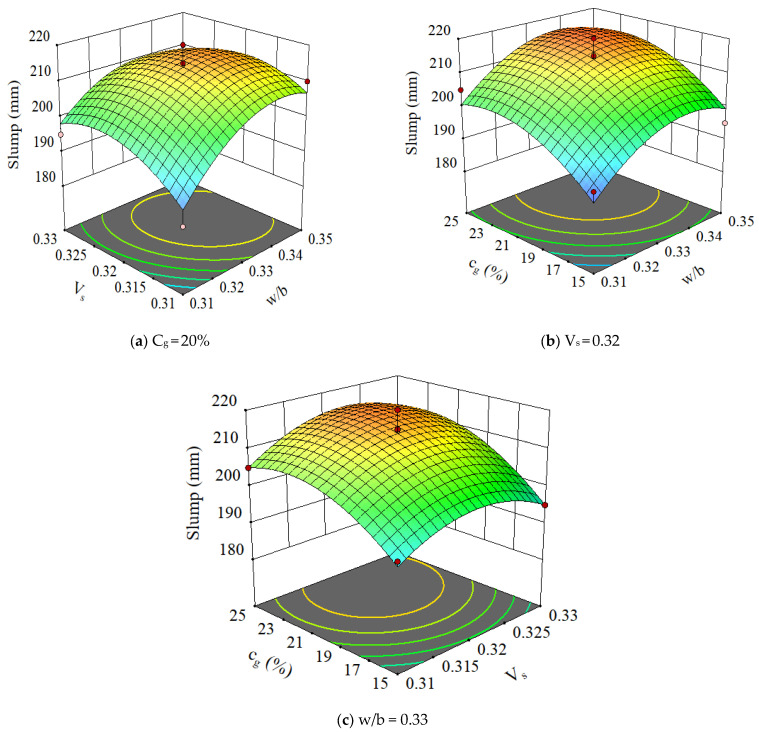
The 3D plots for slump of concrete.

The 3D plots of the slump with variations of various parameters are shown in Figure 5. It can be seen that the slump increased rapidly with w/b before 0.34 and slightly decreased after 0.34 of w/b, indicating a strong correlation between the slump and w/b. The influence of the slurry volume ratio and GLP content on the concrete slump is similar to that of the w/b on the concrete slump. Specifically, as the slurry volume ratio and GLP content increased, the concrete slump initially increased and then decreased. However, the variation range is relatively small, which indicates a weak dependence of the slump flow on slurry volume ratio and content of GLP. It is well acknowledged that w/b significantly influences the slump of concrete mixtures. In the current study, the dosage of the water reducer is fixed, and an additional around 20 kg of water is added per cubic mixture with w/b increasing from 0.31 to 0.35. The excessive water results in slight segregation and bleeding. This is due to the fact that the concrete mixture with limestone powder is easier to segregate and bleed compared to common concrete, which has been observed and investigated by the authors. Therefore, the increase in w/b after 0.34 shows an adverse influence on the slump.

### 4.2. Whiteness

**Figure 6:**
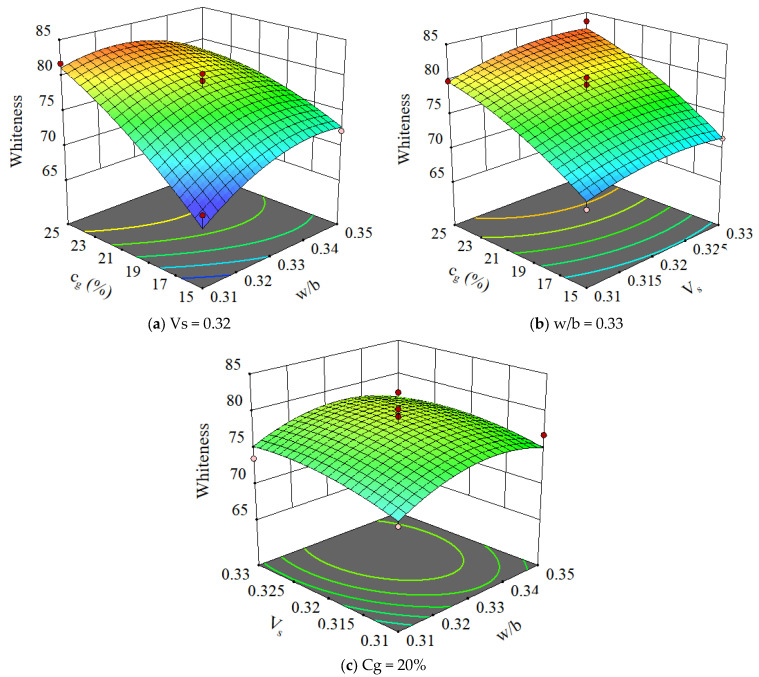
The 3D plots for whiteness of concrete.

The 3D plots of concrete whiteness with variations of various parameters are shown in Figure 6. It can be seen that the w/b and slurry volume ratio had little effect on concrete whiteness. However, the GLP content had a significant effect on concrete whiteness. As the GLP content increased, the concrete whiteness gradually increased. This is attributed to the fact that the whiteness of GLP is 6.6% higher than that of white Portland cement. Moreover, GLP is mainly inert in concrete, which means it is less involved in hydration compared with other components and thus maintains its whiteness.

### 4.3. Compressive Strength

**Figure 7:**
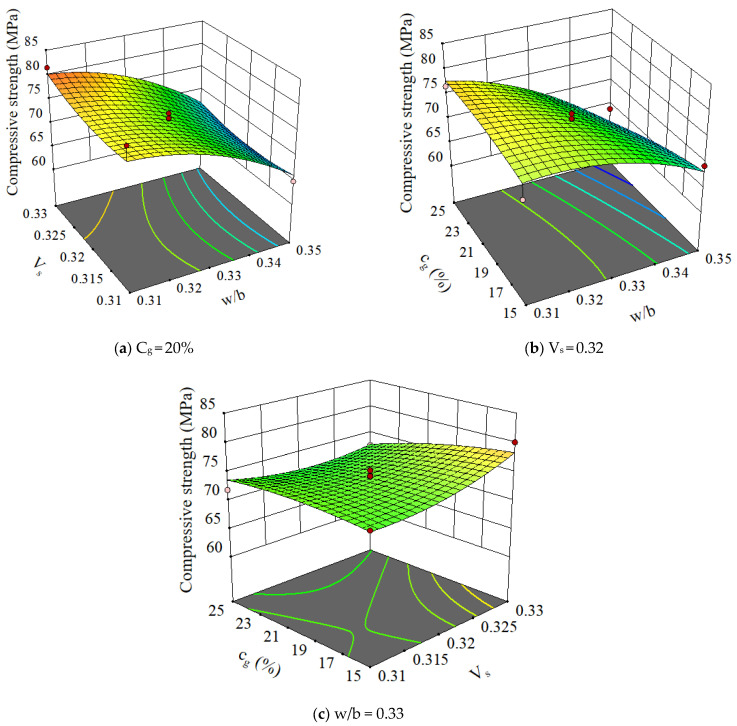
Response surface analysis results for various factors.

The 3D plots of 28-day compressive strength with variations of various parameters are shown in Figure 7. It can be seen that the slurry volume ratio and GLP content have little influence on the compressive strength of concrete. In contrast, the w/b had a prominent influence on the compressive strength of concrete. As the w/b increased, the compressive strength of concrete markedly decreased. This is primarily because an increase in the w/b generates an increase in the volume of concrete mixing water. The excess mixing water evaporates after the concrete hardening, which leaves unexpected pores in the concrete. Thus, the compressive strength of the concrete decreases.

### 4.4. Morphology Analysis

**Figure 8:**
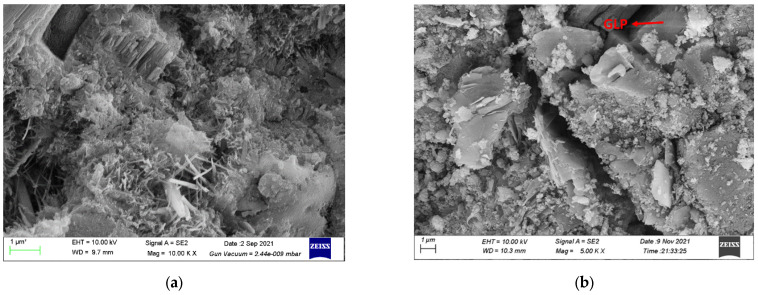
(**a**) Microstructure of white concrete at (**a**) 3 days, and (**b**) 28 days.

The SEM images of concrete mixtures with GLP are shown in Figure 8. It can be seen that GLP particles occupy the pores of cement particles, making the hardened paste denser. The main component of GLP is calcite as shown in Figure 2, providing the filler effect [24,25,26,27], dilution effect [28,29,30], and nucleation effect [31,32,33,34] for concrete. The filler effect of GLP is a result of its small-sized particles filling the gaps between the particles of cementitious material, which can be proven by the particle size distributions of cement and GLP particles shown in Figure 3. This effect is expected to improve the particle accumulation and particle size distribution of cement-based materials, thereby reducing their porosity and increasing their density. The diluting effect of GLP is due to its low reactivity, i.e., a low proportion of GLP participates in the hydration reaction. Thus, its substitution for cement reduces the content of cementitious materials, thus reducing the hydration products of cement-based materials per unit volume. The ability of GLP to act as a nucleus of crystallization is due to calcium carbonate particles being able to adsorb calcium ions released during the hydration of tricalcium silicate (C_3_S). This decreases the concentration and directional arrangement of calcium hydroxide crystals at the interface, which increases the content of C-S-H at the interface, thus providing nucleation sites for hydration products [10,35]. As can be seen from Figure 8, the GLP particles fill between the pores of the cement particles, making the slurry more compact. In addition, the GLP reduces the enrichment and directional arrangement of calcium hydroxide, provides the crystal nucleus for the hydration products, and plays a significant role in the crystal core action.

## 5. Multi-Objective Optimization and Verification

Mixture optimization is performed to determine the optimal mix proportion by maximizing the slump, whiteness, and compressive strength. A target criterion is set up so that all the possible combinations for the variables and responses are captured. The internal optimization program of Design-Expert software is used to optimize the mixing proportion parameters of WHSC. The optimal mixing proportion parameters of WHSC are obtained with a w/b of 0.333, a Vs of 0.326, and a GLP content of 22.4%. Accordingly, a WHSC was prepared with the optimized mix proportion parameters, followed by the measurement of properties with results shown in Table 11. Meanwhile, the prediction was also performed with the relative error calculated by Equation (4), and the results are also shown in Table 11.

It is demonstrated that the relative error between the test value and the predicted value is less than 2%, indicating the favorable predicting performance of the developed model. Therefore, WHSC with preferred multi-objective properties is obtained by the multi-objective optimization of RSM.

(4) $$D=\frac{\left|E-P\right|}{E}\times 100\%$$

where *D* is the relative error, *E* is the experimental value, and *p* is the predicted value.

## 6. Conclusions

In this paper, RSM is used to evaluate and predict the properties of WHSC prepared with GLP. The following conclusions can be drawn from the findings.

- The slump, whiteness, and compressive strength can be well predicted by the Box–Behnken models with high accuracy.
- Both slump and compressive strength of concrete are significantly influenced by w/b while Vs, and C_g_ show moderate influence.
- Increased GLP content significantly improves the whiteness of high-strength concrete while this property is negligibly influenced by w/b and V_s_.
- Multi-objective optimization is performed via RSM to provide optimized mix proportion with the maximized slump, whiteness, and compressive strength. The optimal mix proportion is the w/b of 0.333, Vs of 0.326, and C_g_ of 22.4%.

This paper demonstrates the feasibility of preparing WHSC with GLP, meanwhile presenting the potential of using RSM in mix proportioning of concrete.

## Figures and Tables

**Table 1:** Technical data of white Portland cement.

Specific Surface Area (m^2^/kg)	Whiteness	Specific Density (g/cm^3^)	Setting Time (Min)		Compressive Strength (MPa)	
			Initial Setting	Final Setting	3 Days	28 Days
334	91.1	3.08	172	270	36.5	61.8

**Table 2:** Chemical composition of white Portland cement and GLP (%).

Oxide	CaO	SiO_2_	Al_2_O_3_	Fe_2_O_3_	SO_3_	K_2_O	Na_2_O	MgO	P_2_O_5_
White cement	72.27	18.77	3.98	0.21	3.73	0.43	0.06	0.21	-
GLP	97.57	0.22	0.06	0.04	-	-	-	1.97	0.08

**Table 3:** Factors and factor levels of independent variables for the experimental program.

Independent Variable Factor	Coding and Level		
	−1	0	1
w/b	0.31	0.33	0.35
Vs	0.31	0.32	0.33
Cg (%)	15	20	25

**Table 4:** Mix proportions of concrete.

No.	w/b	Vs	Cg	Cement (kg/m^3^)	GLP (kg/m^3^)	Water (kg/m^3^)	Sand (kg/m^3^)	Coarse Aggregate (kg/m^3^)	Water- Reducing Agent (kg/m^3^)
1	0	0	0	383.6	95.9	158.2	756.8	1045.2	4.78
2	1	−1	0	360.9	90.2	157.9	768.0	1060.5	4.51
3	−1	0	1	369.2	123.1	152.6	756.8	1045.2	4.92
4	1	0	1	347.8	116.0	162.3	756.8	1045.2	4.64
5	0	0	0	383.6	95.9	158.2	756.8	1045.2	4.78
6	0	1	1	369.3	123.1	162.3	745.8	1029.7	4.92
7	−1	0	−1	422.1	74.5	154.0	756.8	1045.2	4.97
8	1	1	0	384.2	96.0	168.1	745.8	1029.7	4.80
9	0	−1	1	347.0	115.7	152.7	768.0	1060.5	4.63
10	0	−1	−1	396.7	70.0	154.0	768.0	1060.5	4.66
11	0	0	0	383.6	95.9	158.2	756.8	1045.2	4.78
12	1	0	−1	397.5	70.1	163.7	756.8	1045.2	4.68
13	0	1	−1	422.2	74.5	163.9	745.8	1029.7	4.97
14	0	0	0	383.6	95.9	158.2	756.8	1045.2	4.78
15	−1	−1	0	383.2	95.8	148.5	768.0	1060.5	4.79
16	−1	1	0	407.9	102.0	158.1	745.8	1029.7	5.10
17	0	0	0	383.6	95.9	158.2	756.8	1045.2	4.78

**Table 5:** Measured (meas.) and predicted (pred.) results.

No.	Independent Variable			Slump (mm)		Whiteness		Compressive Strength (MPa)	
	w/b	Vs	Cg	Meas.	Pred.	Meas.	Pred.	Meas.	Pred.
1	0	0	0	210	214	77.3	78.4	74.3	74.0
2	1	−1	0	210	207	76.8	75.1	64.8	66.1
3	−1	0	1	205	201	81.7	81.1	76.6	77.7
4	1	0	1	210	213	76.8	78.6	63.8	60.7
5	0	0	0	210	214	76.8	78.4	73.8	74.0
6	0	1	1	210	211	83.6	82.5	72.6	72.8
7	−1	0	−1	195	195	71.5	71.6	80.1	78.3
8	1	1	0	210	206	77.3	76.5	64.3	67.2
9	0	−1	1	205	205	79.8	79.7	71.9	73.7
10	0	−1	−1	190	187	68.2	66.4	72.6	75.7
11	0	0	0	215	214	78.3	78.4	75.3	74.0
12	1	0	−1	195	199	72.2	72.8	69.4	68.3
13	0	1	−1	195	194	69.1	70.2	73.8	73.6
14	0	0	0	220	214	80.3	78.4	74.2	74.0
15	−1	−1	0	185	189	71.9	72.6	80.3	77.4
16	−1	1	0	195	198	73.6	75.3	81.5	80.2
17	0	0	0	215	214	79.3	78.4	72.2	74.0

**Table 6:** Comprehensive analysis of various models of slump.

Source *	Sequential *p*-Value	Mismatched *p*-Value	Adjusted *R*^2^	Predicted *R*^2^	Evaluate
Linear	0.0446	0.0654	0.3242	0.1710	
2FI	0.9442	0.0368	0.1528	−0.3885	
Quadratic	0.0112	0.2234	0.7294	−0.2605	Suggested
Cubic	0.2234		0.8244		Aliased

* Linear is the linear regression model.2FI is the 2 factors interaction model; quadratic is the quadratic polynomial model; cubic is the cubic polynomial model.

**Table 7:** Comprehensive analysis of various models of whiteness.

Source	Sequential *p*-Value	Mismatched *p*-Value	Adjusted *R*^2^	Predicted *R*^2^	Evaluate
Linear	0.0006	0.0918	0.6629	0.5316	Suggested
2FI	0.4025	0.0822	0.6688	0.3726	
Quadratic	0.0437	0.1905	0.8221	0.1370	Suggested
Cubic	0.1905		0.8941		Aliased

**Table 8:** Comprehensive analysis of various models of compressive strength.

Source	Sequential *p*-Value	Mismatched *p*-Value	Adjusted *R*^2^	Predicted *R*^2^	Evaluate
Linear	0.0006	0.0194	0.6589	0.4405	Suggested
2FI	0.3721	0.0175	0.6712	0.0297	
Quadratic	0.017	0.254	0.728	−0.7376	Suggested
Cubic	0.017		0.9538		Aliased

**Table 9:** Analysis of variance of simulation equation.

Source	d_f_	Mean Square			F-Value			*p*-Value		
		Y_1_	Y_2_	Y_3_	Y_1_	Y_2_	Y_3_	Y_1_	Y_2_	Y_3_
Model	9	156.15	31.74	43.20	5.79	9.22	5.76	0.0152	0.0039	0.0155
A	1	312.50	7.22	296.46	11.59	2.10	39.51	0.0114	0.1909	0.0004
B	1	28.12	8.61	7.41	1.04	2.50	0.9878	0.3411	0.1578	0.3534
C	1	378.12	209.10	15.13	14.02	60.72	2.02	0.0072	0.0001	0.1986
AB	1	25.00	0.4225	0.7225	0.9272	0.1227	0.0963	0.3677	0.7364	0.7654
AC	1	2.274 × 10^−13^	19.80	23.04	8.432 × 10^−15^	5.75	3.07	1.0000	0.0476	0.1232
BC	1	6.25	0.4900	7.84	0.2318	0.1423	1.04	0.6449	0.7172	0.3407
A^2^	1	244.80	24.25	28.85	9.08	7.04	3.84	0.0196	0.0328	0.0907
B^2^	1	171.12	5.33	8.05	6.35	1.55	1.07	0.0399	0.2536	0.3348
C^2^	1	171.12	6.84	2.32	6.35	1.99	0.3094	0.0399	0.2015	0.5954
Residual	7	26.96	3.44	7.50						
Lack of Fit	3	39.58	5.30	15.81	2.26	2.59	12.42	0.2234	0.1905	0.0170
Pure Error	4	17.50	2.05	1.27						

**Table 10:** ANOVA for developed response models.

Group	Std. Dev.	Mean	*R* ^2^	Adjusted *R*^2^	Predicted *R*^2^	Press	CV (%)	Signal-To-Noise Ratio
Model Y_1_	5.19	204.41	0.8816	0.7294	−0.2605	2009.38	2.54	6.811
Model Y_2_	1.86	76.14	0.9222	0.8221	0.1370	267.33	2.44	11.285
Model Y_3_	2.74	73.03	0.8810	0.7280	−0.7376	766.80	3.75	9.264

**Table 11:** Comparison of predicted values (pred.) and measured values (meas.) after parameter optimization.

w/b	Vs	Cg(%)	Slump (mm)			Whiteness			Compressive Strength (MPa)		
			Pred.	Meas.	D (%)	Pred.	Meas.	D (%)	Pred.	Meas.	D (%)
0.333	0.326	22.4	215.3	214.5	1.80	80.8	80.2	0.87	72.9	73.3	1.22

## Data Availability

The data presented in this study are available on request from the corresponding author.
